# Tuberculosis treatment outcomes of notified cases: trends and determinants of potential unfavourable outcome, France, 2008 to 2014

**DOI:** 10.2807/1560-7917.ES.2020.25.4.1900191

**Published:** 2020-01-30

**Authors:** Jean-Paul Guthmann, Lucie Léon, Delphine Antoine, Daniel Lévy-Bruhl

**Affiliations:** 1 Santé publique France, French national public health agency, Saint-Maurice, France.

**Keywords:** tuberculosis, epidemiology, surveillance, treatment outcome, France

## Abstract

**Background:**

Surveillance of tuberculosis (TB) treatment outcome, for which reporting has been mandatory in France since 2007, is a key component of TB control.

**Aim:**

We aimed to present surveillance data for non-multidrug-resistant (MDR) cases reported between 2008 and 2014, and identify factors associated with potentially unfavourable treatment outcome.

**Methods:**

Patients were classified according to their treatment outcome 12 months after beginning treatment. Poisson regression with a robust error variance was used to investigate factors associated with potentially unfavourable treatment outcome. Missing data were handled using multiple imputation.

**Results:**

A total of 22,526 cases were analysed for treatment outcome. Information available on treatment outcome increased between 2008 (60%) and 2014 (71%) (p < 0.001). During this period, 74.1% of cases completed treatment, increasing from 73.0% in 2008 to 76.9% in 2014 (p < 0.001). This proportion was 74.0% in culture-positive pulmonary cases. Overall, 19.8% of cases had a potentially unfavourable outcome, including lost-to-follow-up, transferred out, still on treatment, death related to TB and interrupted treatment. Potentially unfavourable outcome was significantly associated with TB severity, residing in congregate settings, homelessness, being a smear-positive pulmonary case, being born abroad and residing in France for < 2 years, history of previous anti-TB treatment and age > 85 years.

**Conclusion:**

Monitoring of treatment outcome is improving over time. The increase in treatment completion over time suggests improved case management. However, treatment outcome monitoring needs to be strengthened in cases belonging to population groups where the percentage of unfavourable outcome is the highest and in cases where surveillance data shows poorer documented follow-up.

## Introduction

According to the World Health Organization (WHO), an estimated 10 million people fell ill with tuberculosis (TB) in 2018 [1]. Although substantial differences exist between countries within the European Union (EU) and the European Economic Area (EEA), the overall situation shows a decreasing trend in TB incidence for the area, where the average estimated incidence rate was 11.5 cases per 100,000 population in 2017 [2]. The decrease was also observed in France, where 5,005 cases were reported in 2017 vs 6,714 in 2000, with notification rate of 7.5 cases per 100,000 population [3].

Early detection and prompt management of patients by adequate and complete treatment, and the investigation of contacts potentially exposed to an index case remain the main tools for TB control. Adequate treatment restores the health of the patient, and prevents ongoing transmission of the infection in the community and the development of drug resistance [4]. Assessing patients’ treatment outcome remains essential for TB control because it allows the identification of cases that have not completed treatment and that, if pulmonary, may potentially continue to transmit the infection to their family or other close contacts in the community. The proportion of cases considered to be cured is therefore a key indicator in the evaluation of national TB control programs. Until 2014, the target set by WHO was 85% of treatment success among new sputum smear-positive cases [5]. This objective was updated by WHO in 2014, and is now 90% [6].

In France, treatment outcome monitoring (TOM) was introduced as part of mandatory notification in 2007. Results for cases notified in 2008 [7] and 2009 [8] showed that 69% and 70% of pulmonary cases had completed their treatment, respectively. In this study, we describe the national TOM results among TB patients reported in France between 2008 and 2014, the last year TOM results were available when our analysis was conducted. Our main study objective was to explore the determinants of potentially unfavourable outcome.

## Methods

### Data source

Our analysis was based on mandatory TB data notified to the regional health office, the Agence Régionale de Santé (ARS), in each French administrative region from 2008 to 2014. The data of cases notified in 2008 and 2009 that were already published [7,8] were updated by taking information received after publication into account.

TB is a mandatorily notifiable disease in France. Here, each physician or microbiologist diagnosing TB should report the case to their corresponding ARS using a standardised paper notification form. Twelve months after the start of treatment or after the date of diagnosis, the ARS requests, through a paper form, information from the notifying physician on the patient’s treatment outcome. This form contains the originally collected patient data and allows the physician to add the outcome. Data are entered in an electronic database by each respective ARS, anonymised, coded and then transmitted by email annually to Santé publique France, the French national public health agency. There, regional databases are then compiled into a single national, validated database. Therefore, the current surveillance system in France produces treatment outcome indicators on year n + 2 after cases are notified.

### Tuberculosis definition and treatment outcome categories

The TB definitions used in France are based on international definitions [9,10]. TB cases to be notified include patients with clinical and/or radiological signs compatible with TB and with a clinician decision to treat the patient with a standard anti-TB treatment, regardless of whether or not cases are confirmed by a positive culture for *Mycobacterium tuberculosis* complex. Following the European definition for surveillance [11], pulmonary TB includes TB affecting lung parenchyma, tracheobronchial tree or larynx. Pulmonary cases may be associated with an extra-pulmonary localisation of the disease. Because of an unclear definition of miliary TB in the notification form (to be changed in 2020), some extra-pulmonary cases are reported as miliary. Therefore, miliary TB cases reported were classified as extra-pulmonary for the analysis.

Patients were classified into seven categories based on European recommendations [12] (Table 1). 

**Table 1:** Tuberculosis treatment outcome categories used for surveillance and the analysis, France, 2008–2014

Treatment outcome classification used for surveillance [12]	Treatment outcome definition [12]	Treatment outcome classification used in the analysis
1. Treatment completed	Patient declared cured by a clinician, with or without documented bacteriological conversion, and has taken at least 80% of the standard anti-TB treatment	1. Treatment completed
2. Death	Death during treatment from another cause than TB	2. Death with no link to TB or with no information concerning a possible link with TB
	Unknown link between death during treatment and TB	
	Death during treatment from TB	3. Potentially unfavourable outcome
3. Treatment stopped	Treatment stopped because of a diagnosis other than TB (when the reason of treatment interruption was ‘other diagnosis’ cases were excluded from the analysis)	
4. Transfer out	Patient transferred to another hospital or to another physician than the notifying person	
5. Lost to follow-up	Patient lost-to-follow-up during treatment and remains lost-to-follow-up 12 months after starting treatment	
6. Still on treatment at 12 months	Patient still on treatment at 12 months, for reasons including:	
	Treatment interruption for more than 2 months	
	Treatment change for one or several reasons: initial or acquired non-MDR drug resistance, adverse reactions to treatment, failure of the initial treatment (insufficient clinical response or non-negativity of bacteriological results)	
	Treatment initially planned for more than 12 months	4. Non-evaluable situation
7. Information unknown	NA	Not analysed

NA: not applicable.

The cohort eligible for our analysis excluded patients who, after notification, were found to not have TB, i.e. those with atypical mycobacteria, cancer, etc., and patients who did not start treatment after diagnosis. These are patients who died before beginning treatment, notified as post-mortem diagnosis (n = 470), and patients who were lost before the beginning of treatment (n = 130). In France, because TOM is based on the situation of the patient within 12 months following the start of treatment, cases identified as MDR were excluded from this analysis. This includes cases identified as MDR in the notification record, i.e. resistant to isoniazid and rifampicin, and cases resistant to rifampicin only, often an indicator of MDR TB and recommended by WHO to be treated as such [13]. Cases included in the analysis were further categorised in four groups, a classification adapted from the one usually used internationally [14] (Table 1). Culture results are not always available in the initial notification and rarely available at the end of the treatment; hence, the proportion of patients bacteriologically cured is not presented in this article.

### Statistical analysis of determinants of potentially unfavourable outcome

Two separate analyses were performed. The first was a univariate analysis where we described treatment outcomes for eligible cases where information on treatment outcome was available. The second was a multivariate analysis where missing values in our dataset were handled using multiple imputation (MI) [15]. The association between patients’ characteristics and having a potentially unfavourable outcome was then analysed through Poisson regression with a robust error variance.

#### Univariate analysis

The denominator for calculated percentages was the number of cases with known information. Comparisons were made using the chi-squared test for the comparison of proportions and the Student’s t test for comparisons of means. Variables with a p value of less than 0.2 in the univariable analysis were included in the multivariate analysis. Region of notification (n = 18) were grouped into five large geographical areas: North West, North East, South West, South East regions based on a vertical and horizontal geographical division of France, and Ile-de-France. The latter is usually identified as a single region given that it concentrates ca 20% of the French population and has specific social, demographic and economic characteristics. The overseas regions, which account for a small number of cases and could not be considered in a unique group, were included in the southern regions: Guadeloupe, Martinique and French Guiana were included in the South West region while Reunion and Mayotte were included in the South East region.

#### Multivariate analysis

In a first step, we handled missing values applying MI [15]. The dataset included 21,155 observations, of which 26.7% were complete. The percentage of missing data ranged from 0.0% to 37.5% according to the different variables of interest (Table 2).

**Table 2:** Distribution of missing data, tuberculosis treatment outcomes analysis, France, 2008–2014 (n = 21,155)

Variable	Missing data	
	n	%
Unfavourable outcome	0	0
Region of notification	0	0
Year of notification	0	0
Site, if extra-pulmonary TB	0	0
Age	4	0.02
Sex	92	0.4
Severe TB (meningeal or miliary)	256	1.2
Type of health professional notifying	272	1.3
Residing in a congregate setting	2,428	11.5
Being homeless	2,637	12.5
Being a health or social worker	2,651	12.5
Smear positive pulmonary case	2,806	13.3
Place of birth and year of arrival to France if foreign-born	4,173	19.7
Previous anti-TB treatment	7,655	36.2
Culture at start of treatment	7,938	37.5

TB: tuberculosis.

We estimated missing values using imputation by chained equations using the ice procedure in Stata version 14.2 (Stata Corporation, College Station, United States (US)). This flexible and practical approach is based on a set of imputation models, one for each variable with missing values [15,16]. Each incomplete variable was successively completed using a multivariate model including all possible predictor variables. Among the set of 20 predictor variables, 11 were incomplete. Since there were 73.3% incomplete observations, 80 complete datasets were generated [17].

In a second step, we used a Poisson regression model with a robust error variance to determine factors associated to treatment failure [18]. Relevant interactions found to be significant in the univariate analysis (p < 0.05) were added in the multiple imputation model. Five statistical interactions were included: sex with age, sex with severe TB (meningeal or miliary), sex with the type of health professional responsible notifying, severe TB with being homeless and severe TB with residing in a congregate setting. The regression model was applied separately to the 80 imputed datasets, then applied jointly to obtain global estimates according to Rubin’s rules [19]. A complete-case analysis using the same regression method was also performed on 8,389 observations, corresponding to 39.6% of the entire dataset. All statistical analyses were conducted using Stata version 14.2 . Age was included as a continuous covariate and fractional polynomials were [20] used to estimate the relationship between the outcome variable and age.

### Ethical statement

As part of the mandatory notification system, information that surveillance data may be used for public health purposes should be given to each tuberculosis case when the case is diagnosed and notified. Ethical approval was not needed to analyse these data.

## Results

### Treatment outcome analysis

A total of 36,117 TB cases were reported between 2008 and 2014, among which, 34,762 were eligible for treatment outcome analysis (incidence range/year: 4,691–5,593) (Table 3).

**Table 3:** Tuberculosis cases notified and cases included in the treatment outcome analysis, France, 2008–2014 (n = 36,117)

Cases	Year of notification and number of notified cases														Total	
	2008		2009		2010		2011		2012		2013		2014			
	N = 5,783		N = 5,278		N = 5,218		N = 5,000		N = 5,003		N = 4,947		N = 4,888		N = 36,117	
	n	%	n	%	n	%	n	%	n	%	n	%	n	%	n	%
**Exclusion criteria^a^**	190	3.3	194	3.7	164	3.1	211	4.2	202	4.0	197	4.0	197	4.0	1,355	3.7
Post-mortem diagnosis	86	1.5	46	0.9	79	1.5	82	1.6	61	1.2	64	1.3	56	1.1	474	1.3
TB diagnosis excluded after notification	80	1.4	114	2.2	62	1.2	95	1.9	86	1.7	83	1.7	74	1.5	594	1.6
MDR or RR TB	24	0.4	34	0.6	23	0.4	34	0.7	55	1.1	50	1.0	67	1.4	287	0.8
**Eligible for treatment outcome analysis^a^**	5,593	96.7	5,084	96.3	5,054	96.9	4,789	95.8	4,801	96.0	4,750	96.0	4,691	96.0	34,762	96.2
**No information on treatment outcome^b^**	2,256	40.3	1,920	37.8	1,916	37.9	1,584	33.1	1,762	36.7	1,464	30.8	1,334	28.4	12,236	35.2
**Analysed for treatment outcome^b^**	3,337	59.7	3,164	62.2	3,138	62.1	3,205	66.9	3,039	63.3	3,286	69.2	3,357	71.6	22,526	64.8

MDR: multidrug-resistant; RR: rifampicin-resistant; TB: tuberculosis.

^a^ The denominator for calculation of percentages for the exclusion criteria and eligible for treatment outcome analysis is the number of cases reported (N).

^b^ The denominator for calculation of percentages of both cases with ‘no information on treatment outcome’ and cases ‘analysed for treatment outcome’ is the number of cases eligible for treatment outcome analysis (N−the number of cases excluded).

Of the 36,117, 34,762 (96.2%) were eligible for the treatment outcome analysis and of these, 22,526 (64.8%) had known information on treatment outcome and were thus kept for the analysis. The number of cases with information on treatment outcome increased significantly over time, from 59.7% in 2008 to 71.6% in 2014 (p < 0.001). The number of districts, areas making up regions, that did not provide information on treatment outcome decreased from 8.9% to 3.0% over the period.

The main characteristics of cases with known information on treatment outcome were similar each year except for age (p < 0.001), people born abroad (p < 0.001), cases with a culture result available at the beginning of treatment (p = 0.004) and cases with a positive culture result (p = 0.015) (Table 4).

**Table 4:** Main characteristics of cases included in the analysis and results of tuberculosis treatment outcome, France, 2008–2014 (n = 22,526)

Treatment outcomes and characteristics	Year of notification and number of cases analysed																p^a^
	2008		2009		2010		2011		2012		2013		2014		Total		
	N = 3,337		N = 3,164		N = 3,138		N = 3,205		N = 3,039		N = 3,286		N = 3,357		N = 22,526		
	%	n	%	n	%	n	%	n	%	n	%	n	%	n	%	n	
Treatment completed^b^	73.0	2,435	71.8	2,272	73.7	2,314	73.8	2,366	74.6	2,267	75.1	2,467	76.9	2,580	74.1	16,701	**< 0.001**
Potentially unfavourable outcome^b^	20.3	677	20.9	663	20.6	646	19.6	628	19.1	580	19.8	651	18.2	610	19.8	4,455	0.09
Death with no link to TB (or link unknown)^b^	5.5	185	6.2	194	4.4	135	4.9	159	4.9	148	3.9	129	3.9	132	4.8	1,082	**< 0.001**
Non-evaluable^b^	1.2	40	1.1	35	1.4	43	1.6	52	1.4	44	1.2	39	1.0	35	1.3	288	0.40
**Male** ^c^	59.0	1,965	58.4	1,843	60.4	1,889	59.8	1,907	58.7	1,773	60.7	1,984	61.0	2,040	59.7	13,401	0.19
**Median age in years (IQR)** ^c^	44 (30–64)		44 (29–65)		43 (29–62)		42 (28–62)		42 (29–64)		41 (29–60)		40 (28–61)		42 (29-63)		**< 0.001**
**Born abroad** ^c^	49.0	1,517	50.7	1,492	53.5	1,580	52.8	1,578	55.2	1,557	55.5	1,702	59.2	1,878	53.7	11,304	**< 0.001**
**Previous anti-tuberculosis treatment** ^c^	9.4	213	11.5	235	11.6	231	11.0	222	11.1	210	10.6	2,019	9.4	195	10.6	1,525	0.14
**Pulmonary tuberculosis** ^c^	71.5	2,310	73.3	2,295	72.3	2,190	74.3	2,359	72.6	2,154	72.9	2,384	72.7	2,428	72.8	16,120	0.38
**Smear positive among pulmonary cases** ^c^	53.9	1,178	52.4	1,131	51.4	1,057	51.5	1,141	49.7	1,008	52.1	1,180	50.8	1,172	51.7	7867	0.19
**Culture-positive among cases with a culture result** ^c^	94.4	1,945	94.3	1,812	93.5	1,870	94.2	1,916	93.5	1,746	93.1	1,905	92.9	2,050	93.7	13,244	0.28
**Culture-positive among all cases analysed** ^b^	58.3	1,945	57.3	1,812	59.6	1,870	59.8	1,916	57.4	1,746	57.9	1,905	61.0	2,050	58.8	13,244	**0.015**

IQR: interquartile range; TB: tuberculosis.

^a^ Test for trend: bolded figures indicate statistical significance (p < 0.05).

^b^ For the treatment outcomes and culture-positive among all cases analysed, the denominator for the calculation of percentages is the number of cases analysed (N).

^c^ For all other characteristics of cases, the percentages are calculated using the denominator N−missing values for that specific characteristic.

Cases with (included in the analysis) and without (excluded) known information on treatment outcome were similar for several characteristics: age (p = 0.09), born abroad (p = 0.31), previous anti-tuberculosis treatment (p = 0.25), positive culture result (p = 0.73). However, they were different for other characteristics; compared with the excluded cases, the group of analysed cases had fewer men (59.7% vs 60.9%, p = 0.04) and fewer smear-positive cases (51.7% vs 53.2%, p = 0.03), but more pulmonary cases (72.3% vs 69.0%, p < 0.001) and more cases with a culture result (62.7% vs 44.2%, p < 0.001).

Overall, 74.1% of cases had completed their treatment, 19.8% had a potentially unfavourable outcome, 4.8% had died from a cause not linked to TB (or link unknown) and 1.3% were non-evaluable (Table 4). The proportion of cases completing treatment increased significantly over time (p < 0.001). The proportion of cases with treatment completed was 73.2% (11,941/16,296), 70.6% (5,606/7,939) and 74.0% (7,645/10,338) in pulmonary cases, smear positive pulmonary cases and culture positive pulmonary cases, respectively. The main causes of potentially unfavourable outcome were (overall/pulmonary cases): lost-to-follow-up (44%/45%), transfer-out (25%/25%), still on treatment at 12 months (13%/11%), death from TB (9%/10%), treatment stopped because of other diagnosis or other reason (9%/9%). Losses to follow-up were significantly more frequent in cases born abroad (in France < 2 years: 13.3%; 2–4 years: 10.0%; 5–9 years: 10.2%; ≥ 10 years: 7.9%) than in cases born in France (6.4%) (p < 0.01 for all comparisons). Potentially unfavourable outcome was more frequent in males compared with females (23.3% vs 17.8%, p < 0.001) and increased with age (13.4% in cases < 10 years old vs 16.6% in cases 70–79 years old and 20.4% in cases ≥ 80 years old, p = 0.025). There was a twofold increase of districts that achieved a treatment completion rate > 85%, with 13% achieving such in 2008 to 23.2% in 2014 (p = 0.07). During the same period, the percentage of deaths linked to TB decreased from 1.9% to 1.4%. (p = 0.11).

### Determinants of potentially unfavourable treatment outcome

Because of a significant interaction between sex and age, results are presented in two separate tables by sex. Having a potentially unfavourable treatment outcome was associated with severity of TB, residing in a congregate setting, being homeless, being a smear-positive pulmonary case, being born abroad and residing in France for less than two years, having a history of previous anti-TB treatment and being more than 85 years of age. Being a health or a social worker, notification by a private pneumologist or a clinician of the district TB centre and residing in the north of France decreased the risk of having a potentially unfavourable treatment outcome. This risk significantly decreased with year of notification (Tables 5 and 6).

**Table 5:** Factors associated with male tuberculosis cases having a potential unfavourable outcome, France 2008–2014

Variable	Complete case analysis (n = 5,067)		Multiple imputation analysis (M = 80) (n = 12,596)^a^	
	Prevalence ratio^b^	95% CI	Prevalence ratio^b^	95% CI
**Severe TB (meningeal or miliary)**	**1.52**	1.25–1.86	**1.59**	1.41–1.80
**Increasing year of notification**	**0.96**	0.93–0.98	**0.97**	0.95–0.98
**Region of notification**				
Paris region (Ile de France)	1	Ref	1	Ref
North West	**0.65**	0.55–0.77	**0.67**	0.60–0.74
North East	**0.82**	0.69–0.97	**0.79**	0.71–0.88
South East	0.87	0.73–1.02	0.97	0.88–1.06
South West	**0.76**	0.64–0.91	0.95	0.86–1.05
**Health professional notifying**				
Clinician in hospital	1	Ref	1	Ref
Private pneumologist	**0.67**	0.50–0.88	**0.72**	0.61–0.85
Biologist in hospital	0.89	0.62–1.29	1.08	0.91–1.29
Clinician of district TB centre	1.04	0.85–1.28	**0.84**	0.74–0.97
Other	0.72	0.42–1.24	1.04	0.85–1.29
**Residing in a congregate setting**	**1.33**	1.17–1.52	**1.28**	1.18–1.39
**Being a healthcare or social worker**	0.76	0.55–1.04	**0.76**	0.62–0.92
**Being homeless**	**1.55**	1.32–1.82	**1.54**	1.39–1.70
**Smear positive pulmonary case**	**1.17**	1.06–1.30	**1.20**	1.13–1.28
**Place of birth**				
In France	1	Ref	1	Ref
Abroad, in France ≥ 2 years	0.91	0.80–1.02	0.95	0.87–1.04
Abroad, in France < 2 years	1.11	0.94–1.31	**1.19**	1.06–1.33
**Previous anti-TB treatment**	**1.34**	1.15–1.54	**1.24**	1.11–1.38
**Age (years)**				
5	1.00	0.98–1.03	**1.03**	1.01–1.05
10	1.00	0.99–1.02	**1.01**	1.00–1.02
15	1	Ref	1	Ref
20	1.00	0.98–1.01	**0.98**	0.97–1.00
30	1.00	0.95–1.04	**0.95**	0.92–0.99
40	0.99	0.92–1.06	**0.93**	0.87–0.99
50	0.99	0.90–1.09	**0.91**	0.84–1.00
60	0.99	0.87–1.12	0.92	0.84–1.02
70	0.99	0.84–1.15	0.96	0.87–1.07
75	0.99	0.83–1.16	1.00	0.90–1.11
80	0.99	0.82–1.18	1.04	0.94–1.17
85	0.99	0.80–1.19	**1.12**	1.00–1.26
90	0.99	0.79–1.21	**1.22**	1.05–1.41
95	0.99	0.78–1.22	**1.34**	1.12–1.62

CI: confidence interval; M: number of imputations; Ref: reference group for comparison; TB: tuberculosis.

^a^ Estimated number observations.

^b^ Bolded ratios are statistically significant.

**Table 6:** Factors associated with female tuberculosis cases having a potential unfavourable outcome, France 2008–2014

Variable	Complete case analysis (n = 3,322)		Multiple imputation analysis (M = 80) (n= 8,541)^a^	
	Prevalence ratio^b^	95% CI	Prevalence ratio^b^	95% CI
**Severe TB (meningeal or miliary)**	**1.90**	1.46–2.48	**1.81**	1.53–2.15
**Increasing year of notification**	0.98	0.94–1.02	0.98	0.96–1.00
**Region of notification**				
Paris region (Ile de France)	1	Ref	1	Ref
North West	0.81	0.65–1.02	**0.69**	0.60–0.80
North East	**0.76**	0.58–0.99	**0.73**	0.63–0.85
South East	0.98	0.78–1.23	1.00	0.88–1.14
South West	**0.69**	0.52–0.90	0.87	0.75–1.01
**Health professional notifying**				
Clinician in hospital	1	Ref	1	Ref
Private pneumologist	**0.66**	0.45–0.98	**0.82**	0.68–0.99
Biologist in hospital	**1.68**	1.15–2.45	**1.38**	1.11–1.72
Clinician of district TB centre	0.69	0.48–1.00	**0.68**	0.55–0.85
Other	1.25	0.77–2.05	1.03	0.78–1.36
**Residing in a congregate setting**	1.10	0.84–1.43	**1.21**	1.05–1.40
**Being a health or social worker**	0.82	0.64–1.07	**0.81**	0.68–0.97
**Being homeless**	0.90	0.50–1.63	**1.35**	1.04–1.74
**Smear positive pulmonary case**	**1.19**	1.02–1.40	**1.15**	1.04–1.27
**Place of birth**				
In France	1	Ref	1	Ref
Abroad, in France ≥ 2 years	0.96	0.79–1.17	0.97	0.85–1.10
Abroad, in France < 2 years	**1.36**	1.06–1.74	**1.37**	1.17–1.60
**Previous anti-TB treatment**	1.17	0.90–1.51	**1.33**	1.13–1.56
**Age (years)**				
5	1.00	1.00–1.00	**1.02**	1.00–1.00
10	1.00	1.00–1.00	**1.01**	1.00–1.02
15	1	Ref	1	Ref
20	1.00	1.00–1.01	**0.99**	0.97–1.00
30	**1.02**	1.00–1.03	**0.95**	0.92–0.99
40	**1.06**	1.03–1.08	**0.92**	0.86–0.99
50	**1.11**	1.05–1.18	0.92	0.82–1.02
60	**1.21**	1.10–1.32	0.94	0.83–1.08
70	**1.34**	1.16–1.56	1.04	0.90–1.19
75	**1.44**	1.20–1.73	1.12	0.98–1.29
80	**1.55**	1.25–1.94	**1.24**	1.08–1.42
85	**1.70**	1.30–2.21	**1.41**	1.23–1.63
90	**1.87**	1.37–2.57	**1.66**	1.42–1.95
95	**2.09**	1.44–3.03	**2.03**	1.67–2.47

CI: confidence interval; M: number of imputations; Ref: reference group for comparison; TB: tuberculosis.

^a^ Estimated number observations.

^b^ Bolded ratios are statistically significant.

## Discussion

In this article, we present trends in the treatment outcome of TB cases reported in France over a 7-year period since notification became mandatory in 2007. Although the current surveillance system only allows producing treatment outcome indicators with 2 years of delay, these results are useful in terms of informing tuberculosis policy. In the coming years, the implementation of an electronic-based surveillance system that is currently under development should improve reporting timeliness. Our results suggest improved TOM over time, as shown by both the steady decrease in the number of cases with an unknown treatment outcome and the decrease in the number of districts not providing information on treatment outcome. Although only supported by field observations, several reasons could explain this improvement, such as a greater involvement of TB professionals working in the field, strengthening of the French TB network through periodical meetings where stakeholders are updated with new data and regulations and where they can discuss and exchange field experiences, and the annual dissemination of surveillance results online through the French epidemiological bulletin [21]. These positive results should contribute to encouraging public health professionals in further strengthening of TB surveillance and control.

Our analysis generated higher treatment completion rates than that reported in the EU/EEA countries by WHO/Europe and ECDC [2]. This discrepancy is explained by the exclusion of cases with no information on treatment outcome from our analysis. Unpublished data from regional health authorities and TB centres suggest that this lack of information is mainly because of a lack of reporting rather than insufficient follow-up by the clinician in the field. We decided to exclude these cases because we know that for many of them, the outcome is not reported despite having been ascertained by a clinician. Hence, considering all these cases as not having completed their treatment under-estimates the true completion rate. Regardless, reducing the number of patients with no information on treatment outcome is of crucial importance not only for data reporting coherence, but above all, for better monitoring of the TB control programme in France. We hope that the ongoing shift to an electronic based information system, already partially in place at the regional level, will facilitate a better coordination of the different actors of the TB control network and contribute to this objective.

Our analysis also showed that the percentage of patients who completed their treatment increased over time. Although this increase is small, it suggests improved clinical management and/or better reporting of TOM of TB cases over time both nationally and regionally. This is reinforced by the decrease of deaths linked to TB. In France, surveillance data shows a general decrease in TB incidence [21] and a slight increase in the percentage of patients considered as cured (treatment completed). By decreasing transmission, this last finding could be one of the factors contributing in recent years to the long-term decreasing incidence observed in France. However, the proportion of patients who completed treatment in our study is both below the WHO treatment success rate objective of 90% and the average of 78% for the EU/EEA countries between 2002 and 2011 [14], although different methods of TOM between countries may limit international comparisons [22]. Decreasing the number of losses to follow-up, transfers out and overall deaths (6.5% in our study, which is the average for the EU/EEA countries [14] but above the threshold of 5% considered as acceptable by WHO [12]) should further increase the percentage of TB patients considered cured.

Our study confirmed the association between a potentially unfavourable outcome and several demographic, social and medical factors. The association of this outcome with increasing age, which could be explained by a delayed TB diagnosis and more advanced disease at presentation, was already found by Karo [14]. However, we found that this was only significant in the very elderly (which in our analysis were women ≥ 80 years of age and men ≥ 85 years of age). As in other studies [8,14,23-25], males were at higher risk of having a potentially unfavourable outcome. This could be attributed to behavioural factors such as alcohol consumption or drug use, two factors that are more common among men than women in other European countries [26], but are not included in the French TB surveillance system. Being born abroad was associated with a potentially unfavourable outcome, but this was only the case when arrival in France was recent, i.e. less than 2 years ago. We found the same association in a previous study [8], but the association was with people born abroad and having lived in France less than 10 years before the start of treatment. As underlined by other authors [14,23], foreign origin may be a proxy for other unmeasured characteristics related to migration, such as difficulties speaking and understanding the local language or having a lack of a clear information on how to attend relevant health services. One possible explanation of this association is the higher rate of losses to follow-up among people born abroad, especially those recently arrived to the hosting country, as showed by our results and as observed in the United Kingdom [25]. The reasons for this may be that these individuals may go back to their country of origin or that they may be more reluctant to report information to health professionals because of perceived fear of being deported.

Several disease-related social factors increased the risk of unfavourable outcome in our study. As in our previous study [8], living in congregated settings, including sheltered housing, residential centres, prison or nursing homes was associated with a potentially unfavourable outcome. This was also the case for homelessness, confirming findings from Germany [27]. Conversely, being a healthcare or a social worker significantly decreased the risk of potentially unfavourable outcome. This is possibly because of better awareness of TB and the prevention of it among this group. An association between low risk of unfavourable outcome and residing in the north of France was found in our study, with possible explanation could be an insufficient sensitivity of the mandatory reporting system in France (73%) and its variation among regions [28].

Several factors related to the disease, i.e. severity of the disease, being a smear positive pulmonary case, having a history of anti-TB treatment, independently increased the risk of unfavourable treatment outcome despite these factors likely being related. For example, a severe case may be a smear positive pulmonary case that has been diagnosed with delay, or a case that has discontinued treatment during a previous TB episode and is thus likely to relapse with a more severe form of TB. Finally, being notified by a specialised physician, such as one in a hospital or TB centre, positively impacted treatment outcome. This highlights how maintaining TB capacity is essential for TB control, even in high income countries like France where TB incidence has considerably decreased and is approaching low levels.

Our analysis of surveillance data has several limitations and results should therefore be interpreted with some caution. One limitation is that one third of cases did not have information on treatment outcome, with this group having several different characteristics compared with the group with recorded information on treatment outcome. A possible reason for so many patients missing information on treatment outcome is that patients are often difficult to locate several months after the end of the treatment, either because of an incomplete address in the notification record or because the notifying physician at the hospital level – often a resident who rotates every 6 months as a part of their training – is no longer in charge of the patient and thus unavailable to provide treatment outcome information. Another limitation is that patients who transferred out were classified as having a potentially unfavourable outcome as recommended by WHO. However, when the physician that notified a case in the hospital where the TB diagnosis was initially made receives the request for treatment outcome notification, they may indicate that the patient has been transferred-out to another health structure such as a second-level hospital or ambulatory medicine for care, but this does not necessarily mean that the patient has not been cured. Several field observations show that patients who transfer-out often do complete their treatment, suggesting that our results could underestimate the true percentage of treatment completion in the population of TB patients. Another limitation of our analysis is that patients notified but lost-to-follow-up before the beginning of treatment (e.g. migrants that move and are lost after the notification is made) were not taken into account in our analysis. However, as these represented a very low proportion of the cases notified (n = 110; 0.3% of the cohort), they are unlikely to have had an impact on treatment outcome results. Finally, as the classification of miliary cases was unclear on the reporting form and not always coded as pulmonary, miliary cases were classified as extra-pulmonary in the analysis and could have overestimated the number of cases in this category by a small extent.

One of the strengths of our analysis was the use of MI to analyse the association between factors and potentially unfavourable outcome. With MI, we assumed that the probability of missing data depended on the observed data of one or several covariables. While MI may be unstable for higher rates of missing data [29], this did not seem to be the case in our analysis. This was shown, for example, by the similar distribution of each variable before and after imputation, the diagnostic of the MI, the performance of indicators that were checked (such as relative efficiencies and variance information about MI estimates), and the statistical and epidemiological coherence of results when comparing the MI analysis with the complete case analysis. This allowed us to use the complete dataset of 21,155 observations instead of 8,389 completed observations, providing more accurate estimates of treatment outcome determinants. Furthermore, some variables became statistically significant after MI, e.g. being a healthcare or social worker, place of birth for males or being homeless for females. One limitation was that we were not able to investigate several factors that were not recorded in our surveillance system but have been found to be risk factors in other studies, including being a smoker [30], having diabetes [31,32] or HIV infection [33].

In conclusion, case management and follow-up of TB patients needs to be strengthened, primarily in those belonging to population groups where the percentage of unfavourable outcome is the highest and those where surveillance data shows less well-documented follow up. The electronic-based TB surveillance system that is currently being implemented in France, together with information and training activities for professionals using this new tool, should contribute to improving the monitoring of follow-up of cases. This is particularly because the internet platform will be readily accessible to all actors within the surveillance network thereby contributing to decreasing losses-to-follow-up and allowing timely tracing of transfer-outs. Our study provides information that could help in terms of targeting control measures and strengthening surveillance in the groups with the most need. Our results could help clinicians and other public health professionals involved in TB management adapt case management on at risk groups given what it shows about factors that lead to lower treatment success.
